# The Psychophysiological Effects of Different Tempo Music on Endurance Versus High-Intensity Performances

**DOI:** 10.3389/fpsyg.2020.00074

**Published:** 2020-02-05

**Authors:** Vittoria Maria Patania, Johnny Padulo, Enzo Iuliano, Luca Paolo Ardigò, Dražen Čular, Alen Miletić, Andrea De Giorgio

**Affiliations:** ^1^Faculty of Kinesiology, University of Split, Split, Croatia; ^2^Department of Biomedical Sciences for Health, University of Milan, Milan, Italy; ^3^Faculty of Psychology, eCampus University, Novedrate, Italy; ^4^School of Exercise and Sport Science, Department of Neurosciences, Biomedicine and Movement Sciences, University of Verona, Verona, Italy

**Keywords:** training and testing, rating perception effort, exercise, RPE, sport

## Abstract

The use of music during training represents a special paradigm for trainers to stimulate people undertaking different types of exercise. However, the relationship between the tempo of music and perception of effort during different metabolic demands is still unclear. Therefore, the aim of this research was to determine whether high intensity exercise is more sensitive to the beneficial effects of music than endurance exercise. This study assessed 19 active women (age 26.4 ± 2.6 years) during endurance (walking for 10′ at 6.5 km/h on a treadmill) and high intensity (80% on 1-RM) exercise under four different randomly assigned conditions: no music (NM), with music at 90–110 bpm (LOW), with music at 130–150 bpm (MED), and with music at 170–190 bpm (HIGH). During each trial, heart rate (HR) and the rating of perceived exertion (RPE) were assessed. Repeated analysis of variance measures was used to detect any differences between the four conditions during high intensity and low intensity exercise. RPE showed more substantial changes during the endurance exercises (11%), than during high intensity exercise (6.5%), between HIGH and NM conditions. The metabolic demand during the walking exercise increased between NM and HIGH bpm conditions. This study indicates the benefits of music under stress conditions as well as during endurance and high intensity training. The results demonstrate that the beneficial effects of music are more likely to be seen in endurance exercise. Consequently, music may be considered an important tool to stimulate people engaging in low intensity physical exercise.

## Introduction

The psychophysiological effects of music have been widely investigated in both psychology (De Giorgio et al., 2017; Benke et al., 2018; Innes et al., 2018; Padovan et al., 2018) and in exercise and sport related fields (Jarraya et al., 2012; Terry et al., 2012). The effects of exercise on the brain are well known (for a review see De Giorgio, 2017, De Giorgio et al., 2018a), but the effect that music has on exercise and its cerebral counterpart has only recently been investigated (Bigliassi et al., 2017; Tabei et al., 2017). It has been demonstrated that music is able to trigger behavioral changes, i.e., there is an underlying modification of brain function which can induce people to increase their exercise adherence and participation (Altenmüller and Schlaug, 2012). Music has also been demonstrated to be effective in reducing fatigue and its related symptoms (Jing and Xudong, 2008), in emotional regulation (Hou et al., 2017), in regulating affective arousal (Hutchinson et al., 2018) and in improving the efficacy of the motor system (Bigliassi et al., 2017).

However, music remains a subjective experience and De Nora (2000) discussed how people select music in a subjective manner to improve mood and energy levels during physical activity. Furthermore, the influence of music is also associated with both its intrinsic elements, such as rhythm and musicality and extrinsic factors emerging from cultural and extra-musical associations (Zatorre et al., 2007; Koc and Curtseit, 2009; Karageorghis et al., 2012). Listening to a particular type of music has been found to improve subjective experience during low, moderate, and high intensity exercise (De Nora, 2000; Karageorghis and Priest, 2012a, b; Karageorghis et al., 2012).

In the neurophysiological context, it has been demonstrated that music influences processes in the autonomic nervous system and can even be used to regulate blood pressure and heart rate (HR) (Schneck and Berger, 2006; Karageorghis and Priest, 2012a, b). The central nervous system is highly sensitive to musical cues and its reaction is diverse, involving muscle activation, attention, thoughts, behavior, and executive functions (Thaut, 2005; Thaut and Abiru, 2010; Altenmüller and Schlaug, 2013).

With respect to attention, listening to music during physical activity has been described in the literature as a dissociative cognitive strategy that enables a shift in attention away from subjective experiences of discomfort or pain (De Nora, 2000; Rodriguez-Fornells et al., 2012; Altenmüller and Schlaug, 2013). It has been shown that as the intensity of exercise increases, discomfort and related bodily sensations increase, eliciting a greater awareness of fatigue-related symptoms (Karageorghis and Priest, 2012a). Conversely, when people are exposed to environmental sensory cues such as music, colors, or videos, these cues can divert attention and modify both behavior and bodily discomfort sensations during exercise or other tasks (Karageorghis and Jones, 2014; De Giorgio et al., 2018a, b).

As mentioned previously, the literature describes the capacity of music to shift the focus away from feelings of discomfort and fatigue and this has been demonstrated through the assessment of the rating of perceived exertion (RPE). In particular, it has been found that reduced RPE with music is associated with low to moderate intensity exercise, but not high intensity exercise (Harmon and Kravitz, 2007; Karageorghis and Priest, 2012b). The authors proposed that music seems unable to divert attention during exercise that is overly intense with a high degree of bodily discomfort (for a review see Karageorghis and Priest, 2012a, b). Despite this, it was found that while the RPE during high intensity exercise remained the same with or no music (NM), participants experienced more positive mood profiles when listening to music, regardless of exercise intensity. The authors suggest that this could have occurred because although the participants knew that they were exercising hard, they were happier about the activity (Karageorghis and Priest, 2012a, b). In their literature review, Karageorghis and Priest (2012a, b) also highlighted the finding that trained athletes are less influenced by the effects of music compared to those who are untrained. This could be a result of the practice athletes have in diverting their attention away from bodily discomfort in any situation.

However, to the best of our knowledge there have been no studies to date that have linked the effect of two different exercise types on the RPE under different music conditions. This study investigated the RPE after low intensity and high intensity exercise, conducted under different music conditions.

### Participants

Nineteen female participants ranging from 24 to 31 years old were enrolled for the present study. All participants regularly performed physical activity three to five times a week and a good proportion of the participants were involved in physical fitness. The following participant information was collected: mass, height, BMI (body mass index, obtained by dividing the weight in kg of the participant with the square of the height expressed in meters), training experience [endurance intensive effort and/or high intensity effort (Chamari and Padulo, 2015)], and maximal HR. The participants’ data are reported in Table 1. All participants gave written informed consent following verbal and written explanations regarding the study. All methodological procedures were approved by the local Ethics Committee.

**TABLE 1:** Characteristics of participants.

	Mass (kg)	Height (cm)	BMI (kg/m^2^)	Age (years)	Theoretical maximal HR (bpm)	Training experience	Training experience (years)
Participant 1	67.2	175	22.0	24	187	EIE	16
Participant 2	61.3	168	21.7	26	186	EIE	13
Participant 3	63.8	170	22.1	24	190	EIE	12
Participant 4	67.5	177	21.6	26	175	EIE	15
Participant 5	56.6	167	20.3	29	186	HIE	7
Participant 6	63.9	175	20.9	31	184	EIE	3
Participant 7	62.0	173	20.3	25	185	EIE	14
Participant 8	48.9	160	19.1	24	179	HIE	2
Participant 9	61.3	169	21.5	24	177	EIE	1
Participant 10	57.4	165	21.1	24	190	HIE	5
Participant 11	53.0	170	18.3	24	170	HIE	1
Participant 12	62.9	164	23.4	25	185	HIE	3
Participant 13	70.0	170	24.2	29	184	EIE	11
Participant 14	53.7	170	18.6	31	187	HIE	7
Participant 15	61.3	157	24.9	27	187	HIE	0.6
Participant 16	57.3	177	18.3	31	178	EIE	2
Participant 17	54.5	170	18.9	27	184	HIE	1
Participant 18	55.0	168	19.5	26	184	EIE	4
Participant 19	54.5	173	18.2	25	188	EIE	3
MEAN	59.58	169.37	20.78	26.42	183.47	–	6.35
SD	5.65	5.30	2.02	2.57	5.31	–	5.39

*Mean and standard deviation (SD) for the anthropometric measures and the training experience. EIE, endurance intensive efforts; HIE, high intensity efforts.*

### Socio-Demographic Variables and Enrolment Process

The participants were enrolled in different fitness centers located in Rome using a convenience sampling based on the following inclusion criteria: (1) female gender; (2) age between 18 and 35 years old; (3) at least 1 year of experience in fitness training (minimum three to five session of training per week; and (4) at least high school graduation. To assure the safety of the procedure and the correct interpretation of the data the following exclusion criteria were instead applied: (1) presence of relevant disease or other condition (temporary or permanent) incompatible with the proposed interventions; (2) history of relevant cardiopulmonary disease; (3) BMI > 25 kg/m^2^; (4) presence of relevant disease or other condition (temporary or permanent) potentially influencing the physical performance of the participant. The participants were all volunteers and were invited for an individual appointment in a Sports Science Laboratory in order to explain them the procedure of the study and in order to collect the information via survey. The survey aimed to investigate and collect the data reported in Table 1, the adherence to the inclusion criteria, and the absence of exclusion criteria.

### Procedures

Each participant was asked to perform two different training sessions: (1) walking at 6.5 km/h (endurance exercise) on a treadmill for 10 min to reach a steady state (Padulo et al., 2014) and (2) high intensity exercise on leg press machine based on the one repetition maximum test (1-RM) (Padulo et al., 2015). Each exercise test was performed four times by each participant under four different tempo music conditions. The order of the four music conditions was randomly assigned in a counterbalanced way. Subsequently, in the other four experimental sessions (each conducted under different music conditions) each participant individually performed the two exercise sessions. The participant’s HR was recorded during each endurance session (10 min for each one) and at the end of the test, the participant’s average HR and peak HR were then calculated. Furthermore, immediately after the walking exercise, the participant was asked to express their perception of fatigue as a value based on the Borg scale (0 < 20). On a different day, each participant was assessed with regard to their maximal repetitions during the leg-press exercise (Padulo et al., 2017; Migliaccio et al., 2018) and the 1-RM based on the Brzycki method was calculated at the same time (Padulo et al., 2015). To ensure standardization of the procedure the following method was used. The participant began the leg-press exercise with a load equivalent to their body weight (measured in the preliminary session) and performed the maximal number of repetitions. When the participant reached 10 repetitions with the selected weight, the exercise was stopped and 20 kg was added for a further attempt after four minutes of rest. When the participant was unable to perform more than 10 repetitions with the selected load, the respective 1-RM was calculated using the Brzycki equation: 1-RM theoretical = lifted load/[1.0278 − (0.0278 × repetitions performed)]. Immediately after the last try, the participant was once again asked to express their perception of fatigue as a value based on the Borg scale (CR 0 < 20). The four tempo music conditions were: NM, with music at 90–110 bpm (LOW), with music at 130–150 bpm (MED), and with music at 170–190 bpm (HIGH). During each music condition, five pop songs were used with increasing bpm (e.g., in the MED condition, the first song had 132 bpm, the second had 136 bpm, the third had 141 bpm, the fourth had 143 bpm, and the last song had 148 bpm). All sessions were conducted in the same Sports Science Laboratory of the enrolment process, and similar environmental conditions at each session were ensured (temperature and relative humidity for each session ranging from 22 to 24°C and 25 to 27%, respectively). All the sessions under different music condition were performed one week apart each other to avoid the influence of fatigue of each session on the subsequent ones. A reminder was sent to each participant 2 days before each session in order to assure the correct adherence to the study planning. No dropout or unavailability of the participants occurred during the study.

### Statistical Analysis

The Shapiro–Wilk test was used to evaluate the normality of the data distribution. Successively, multivariate analysis of variance with repeated measures (RM-MANOVA) was conducted to determine whether significant differences existed between the four different music conditions. This was considered as the factor of the analysis (named CONDITION). The following five variables were considered dependent variables: aHRwalking (average HR during walking at 6.5 km/h); pHRwalking (peak HR during walking at 6.5 km/h); RPEwalking (RPE during walking at 6.5 km/h); 1-RMlp (one-repetition maximum during leg-press); and RPElp (RPE during leg-press). The alpha test level for statistical significance was set at *p* < 0.05 and $\eta_{\text{p}}^{2}$ was calculated as the index of effect size. The Bonferroni correction was used for pairwise comparison of the four music conditions. The reliability of the external load time (10′ on treadmill/leg-press machine) was assessed by calculating the intra-class correlations coefficient (ICC), according to the literature (Weir, 2005). The SPSS statistical software package (Version 25.0; IBM) was used for all statistical analysis.

## Results

**FIGURE 1:**
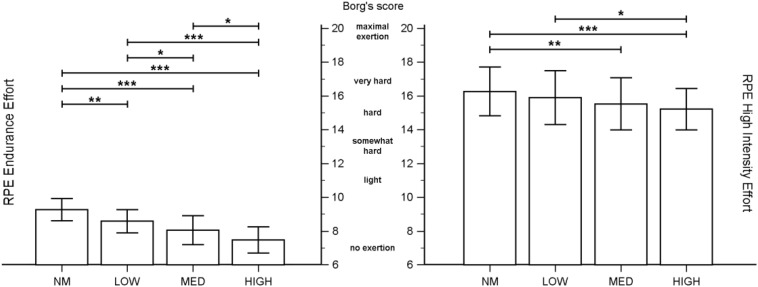
Rating of perceived exertion (RPE) in both exercises during the four conditions. *p < 0.05; **p < 0.01; ***p < 0.001.

On leg-press exercise the 1-RM was 133.26 ± 41.78 kg with the starting load of 62.11 ± 4.19 kg and concluded with 4.21 ± 1.90 sets/3.32 ± 0.89 reps. The ICC for external load time (treadmill/leg-press) on the four conditions was >0.985 for high intensity and endurance exercises, respectively. The results of the RM-MANOVA indicated significant differences between the four conditions (*F*_13_,_6_ = 94,152; *p* < 0.0001; $\eta_{\text{p}}^{2}=0.995$). The univariate analysis showed significant differences with regard to all five dependent variables analyzed: aHRwalking (*F*_3_,_54_ = 242.08; *p* < 0.0001; $\eta_{\text{p}}^{2}=0.931$); pHRwalking (*F*_3_,_54_ = 631.38; *p* < 0.0001; $\eta_{\text{p}}^{2}=0.972$); RPEwalking (*F*_3_,_54_ = 35.27; *p* < 0.0001; $\eta_{\text{p}}^{2}=0.662$); 1-RMlp (*F*_3_,_54_ = 39.54; *p* < 0.0001; $\eta_{\text{p}}^{2}=0.687$); and RPElp (*F*_3_,_54_ = 15.86; *p* < 0.0001; $\eta_{\text{p}}^{2}=0.468$). The results obtained for each condition and the pairwise comparisons between the four conditions are reported in Table 2 and Figure 1.

**TABLE 2:** Results with pairwise comparisons among the four music conditions.

Dependent variables	Music conditions	Mean	SD	95% CI		Pairwise comparisons			
				Lower	Upper	NM vs.	LOW vs.	MED vs.	HIGH vs.
aHRwalking (bpm)	NM	83.37	4.166	81.360	85.376	LOW: *p* < 0.0001	NM: *p* < 0.0001	NM: *p* < 0.0001	NM: *p* < 0.0001
	LOW	95.79	4.237	93.747	97.832	MED: *p* < 0.0001	MED: *p* = 0.001	LOW: *p* = 0.001	LOW: *p* < 0.0001
	MED	99.47	3.289	97.888	101.059	HIGH: *p* < 0.0001	HIGH: *p* < 0.0001	HIGH: *p* < 0.0001	MED: *p* < 0.0001
	HIGH	110.11	4.054	108.151	112.059				
pHRwalking (bpm)	NM	95.95	4.731	93.667	98.228	LOW: *p* < 0.0001	NM: *p* < 0.0001	NM: *p* < 0.0001	NM: *p* < 0.0001
	LOW	104.37	4.044	102.419	106.318	MED: *p* < 0.0001	MED: *p* < 0.0001	LOW: *p* < 0.0001	LOW: *p* < 0.0001
	MED	109.05	4.020	107.115	110.990	HIGH: *p* < 0.0001	HIGH: *p* < 0.0001	HIGH: *p* < 0.0001	MED: *p* < 0.0001
	HIGH	125.37	4.044	123.419	127.318				
RPEwalking (Borg’s scale score)	NM	9.26	0.653	8.948	9.578	LOW: *p* = 0.002	NM: *p* = 0.002	NM: *p* < 0.0001	NM: *p* < 0.0001
	LOW	8.58	0.692	8.245	8.913	MED: *p* < 0.0001	MED: *p* = 0.049	LOW: *p* = 0.049	LOW: *p* < 0.0001
	MED	8.05	0.848	7.644	8.461	HIGH: *p* < 0.0001	HIGH: *p* < 0.0001	HIGH: *p* = 0.045	MED: *p* = 0.045
	HIGH	7.47	0.772	7.101	7.846				
1RMlp (kg)	NM	133.26	41.777	113.127	153.399	LOW: *p* not sign.	NM: *p* not sign.	NM: *p* not sign.	NM: *p* not sign.
	LOW	133.26	41.777	113.127	153.399	MED: *p* not sign.	MED: *p* not sign.	LOW: *p* not sign.	LOW: *p* not sign.
	MED	133.26	41.777	113.127	153.399	HIGH: *p* < 0.0001	HIGH: *p* < 0.0001	HIGH: *p* < 0.0001	MED: *p* not sign.
	HIGH	136.47	43.004	115.746	157.201				
RPElp (Borg’s scale score)	NM	16.26	1.447	15.566	16.961	LOW: *p* not sign.	NM: *p* not sign.	NM: *p* = 0.002	NM: *p* = 0.002
	LOW	15.89	1.595	15.126	16.663	MED: *p* = 0.002	MED: *p* not sign.	LOW: *p* not sign.	LOW: *p* = 0.011
	MED	15.53	1.541	14.784	16.269	HIGH: *p* < 0.0001	HIGH: *p* = 0.011	HIGH: *p* not sign.	MED: *p* not sign.
	HIGH	15.21	1.228	14.618	15.803				

*Mean and standard deviation (SD) and 95% confidence interval (95% CI) for the aHRwalking (average HR during walking at 6.5 km/h); pHRwalking (peak of HR during walking at 6.5 km/h); RPEwalking (RPE during walking at 6.5 km/h); 1RMlp (One-repetition maximum at leg press); and RPElp (RPE during 1-RM at leg press) on four conditions: NM (no music), LOW (music with bpm between 90 and 110), MED (music with bpm between 130 and 150), and HIGH (music with bpm between 170 and 190).*

## Discussion

The results of this study support the findings of other studies regarding the effects of music on cognitive motor processes (Bianco et al., 2017; Mohammad Alipour et al., 2019). This study is the first to link the effect of music with RPE and HR under different music conditions. The effects of music involve both unconscious sub-cortical areas and conscious cortical stimulation and the responses are highly complex (Zatorre et al., 2007; Levitin and Tirovolas, 2009; Rodriguez-Fornells et al., 2012; Altenmüller and Schlaug, 2013). Music can stimulate the human brain to such an extent that to ignore it is more difficult than interaction (Zatorre et al., 2007; Levitin and Tirovolas, 2009).

As mentioned previously, music perception involves both cortical and sub-cortical areas, but it has an effect on the whole brain. Music influences emotional responses (i.e., the limbic system), associate/automatic movements (i.e., the basal ganglia), coordination (i.e., the cerebellum), and the organization and planning of movements (motor, pre-motor, and supplementary motor areas). The rhythmic patterns of music facilitate error correction and the execution of movements (Levitin and Tirovolas, 2009). Indeed, repeated movements seem to be related to the phases between pulse music beats, stimulating a feedback/forward loop (Todd et al., 2002; Levitin and Tirovolas, 2009). In addition to the involvement of the whole brain, music also affects the whole body and this influence occurs through physiological arousal mediated by sub-cortical structures and bodily rhythms such as walking, breathing, and HR (Schneck and Berger, 2006; Levitin and Tirovolas, 2009; Altenmüller and Schlaug, 2013). Previous studies have demonstrated that music regulates processes in the autonomic nervous system and can be used to regulate the cardiovascular system with regard to both HR and blood pressure (Harmon and Kravitz, 2007; Murrock and Higgins, 2009; Karageorghis and Priest, 2012a, b). Bodily activation is very important in feeling fatigue, as signals traveling from the body toward the brain inform the latter on the effort in progress, modulating physical activity as a result. These signals capture conscious attention and can change behavioral responses relating to exercise adherence. Becomes These signals capture conscious attention and can change behavioral responses (De Giorgio, 2016) also relating to exercise adherence. However, music can be considered a useful tool in regulating the intensity of physiological arousal and subjective experiences in order to improve levels of physical activity and exercise participation (De Nora, 2000; Zatorre et al., 2007; Karageorghis and Priest, 2012a, b; Altenmüller and Schlaug, 2013). Indeed, in the context of sport and exercise performance, De Nora (2000) discussed how music can be strategically chosen in order to induce physio-psychological responses that lead to better performance, experience, and adherence to exercise as well as regulating mood and shifting attention away from discomfort (De Nora, 2000). Our investigation showed the differences in the effects of listening to music during high intensity and low intensity exercise (i.e., endurance exercise). Endurance exercise seems more sensitive to external stimuli (Van Cutsem et al., 2017) due to the mental fatigue and perception of effort involved in endurance exercise. High intensity training (i.e., explosive effort) seems characterized by an all-out approach that is powered primarily by metabolic pathways through muscular simulation without the use of oxygen (Van Cutsem et al., 2017). As such, “anaerobic” high intensity training requires fewer decision-making processes compared to endurance exercise, due to the all-out strategy and the intrinsically shorter duration (Van Cutsem et al., 2017).

This study presents some limitations. First, the results refer to a physically trained adult female population. Consequently, these results need to be confirmed for other populations such as male subjects, untrained people, older people, or adolescents. Furthermore, music cannot be described only using tempo, but also other characteristics need to be considered such as lyrics, melody, and genre. These characteristics were not considered in this study, but they could influence the performance of the participant. Also, the preference of the participants concerning their musical preferences were not collected and considered in the present study. Finally, the effect in the different moments of the same exercise was not considered as in previous study (Di Cagno et al., 2016).

## Conclusion

This study indicates the benefits of listening to music under physical stress conditions as well as during endurance and high intensity training. The results of this study demonstrate that the beneficial effects of music are more apparent for endurance exercise. Consequently, music may be considered an important tool to stimulate people engaging in physical exercise. The finding of this study underlines the efficacity of the tempo of music in improving the performance and simultaneously reducing the RPE during the exercises. With this in mind, it is important to understand how this music influence can be used to improve training load and performance in trained people, but also the risk of an “altered” RPE during the exercise (both endurance and high intensity) needs to be clarified.

## Data Availability Statement

The datasets generated for this study are available on request to the corresponding author.

## Ethics Statement

The studies involving human participants were reviewed and approved by the local Ethics Committee. The patients/participants provided their written informed consent to participate in this study.

## Author Contributions

VP, JP, and AD: conceptualization and writing. JP and EI: methodology and formal analysis. EI and LA: validation. DČ, AM, and VP: investigation. LA, DČ, and AM: resources. EI: data curation. AD and JP: review and editing. AD: supervision.

## Conflict of Interest

The authors declare that the research was conducted in the absence of any commercial or financial relationships that could be construed as a potential conflict of interest.
